# Investigation of HoxB3 and Growth Factors Expression in Placentas of Various Gestational Ages

**DOI:** 10.3390/jdb10010002

**Published:** 2021-12-23

**Authors:** Ilze Kreicberga, Anna Junga, Māra Pilmane

**Affiliations:** Institute of Anatomy and Anthropology, Riga Stradins University, Kronvalda Bulv. 9, LV-1010 Riga, Latvia; Ilze.Kreicberga@rsu.lv (I.K.); Mara.Pilmane@rsu.lv (M.P.)

**Keywords:** gene, immunocytochemistry, microscopy, development, fetus, growth, intrauterine growth, placenta

## Abstract

An evaluation of transforming growth factor beta (TGFβ), hepatocyte growth factor (HGF), basic fibroblast growth factor (FGF-2), fibroblast growth factors receptor 1 (FGFR1) and Hox-positive cells in the human placenta, and their correlation with gestational time at delivery and pregnancy outcomes, may provide not only a better understanding of the role of Hox genes and growth factors in human development, but also may be of clinical importance in reproductive medicine. This study analyzed the immunohistochemical identification of TGFβ, HGF, FGF-2, FGFR1 and HoxB3 in placentas of various gestational ages. We found few (+) TGFβ, moderate (++) FGF-2 and numerous (+++) HGF and FGFR1 positive structures. Occasional (0/+) to numerous (+++) HoxB3-positive structures were detected in different types of placental cells specifically, cytotrophoblasts, syncytiotrophoblast, extravillous trophoblasts, and Höfbauer cells. Correlating the appearance of HoxB3 staining in placentas with neonatal parameters, we found a statistically significant negative correlation with ponderal index (r = −0.323, *p* = 0.018) and positive correlation with neonate body length (r = 0.541, *p* = 0.046). The number of HoxB3-positive cells did not correlate with growth factors and gestational age, but with neonatal anthropometrical parameters, indicating the role of HoxB3 not only in placental development, but also in the longitudinal growth of the fetus. TGFβ and FGF-2 did not play a significant role in the development of the placenta beyond 22nd week of pregnancy, while HGF and FGFR1 immunoreactive cells increased with advancing gestation, indicating increasingly evolving maturation (growth, proliferation) of the placenta, especially in the third trimester.

## 1. Introduction

Morphogenesis of the developing human embryo is determined by numerous homeobox (Hox) genes, a large family of similar genes that direct the formation of many body structures during early embryonic development [1]. Originally discovered in Drosophila melanogaster as determinants of the anterior-posterior axis, members of the Hox family have determinant roles in the development of vertebrates. The Hox gene cluster has given rise to 39 genes in mammals, separated into four separate chromosomal clusters designated Hox A, B, C and D [1,2].

Homeobox genes are known to regulate stem cell differentiation and encode anatomic segment identity, that is, whether the embryo will form with bilateral symmetry with a head (anterior), tail (posterior), back (dorsal), and belly (ventral) [3]. Hox genes are directly or indirectly involved in a variety of developmental disorders, diseases and cancers [4,5,6]. The dysregulation of HoxB3 is associated with a number of malignancies including leukemia [2], endometrial cancer [7], glioblastoma [8], colon cancer [9], lung adenocarcinoma [10], and breast cancer [11].

Hox proteins have been studied in different fields of human medicine and play an important role in human reproduction. Members of the Hox family have proven roles in the development of the female genital tract [12], and changes in their expression can lead to endometriosis and infertility [7,13,14]. Hox proteins ensure implantation and decidualization [15,16]. Further, they are involved in the maintenance of certain types of trophoblast cells [17] and are needed for the differentiation of cytotrophoblasts into syncytiotrophoblasts [18]. In addition, a decrease of HoxB3 might inhibit the invasion of trophoblast cells [6]. HoxB3 has a certain significance for the risk evaluation of pre-eclampsia [19]. HoxB3 promotes invasive behaviour of endothelial cells in response to angiogenic stimulation [1] and is required for the morphogenesis of new capillary tubes [20].

The transforming growth factor beta (TGFβ) is a vital regulator of placental functions [21]. TGFβ is known to regulate a variety of cellular functions, including cell proliferation, differentiation, apoptosis, migration/invasion, matrix synthesis and immune response [22].

Hepatocyte growth factor (HGF), also named “hepatotropin”, is a heterodimer molecule composed of a 69 kDa alpha-subunit and a 34 kDa beta-subunit [23]. HGF is a multifunctional growth factor and is expressed in a wide variety of cells, including the cytotrophoblast of the placenta. It seems to play a significant role in the development of the placenta [24].

Basic fibroblast growth factor (FGF-2) is a member of the family of fibroblast growth factors known to have impacts on vasculogenesis, wound healing and embryogenesis [25]. The expression of FGF-2 in the placental tissues gradually decreases with the development of the placenta [26]. FGF-2 exerts proangiogenic actions via activation of fibroblast growth factor receptor 1 (FGFR1) [27]. FGF-2 activates FGFR1 in cooperation with heparin or heparin sulfate proteoglycan [28]. The FGF/FGFR system has been implicated in a variety of physiological and pathological conditions. These include embryonic development, tissue growth and remodeling, inflammation, tumor growth, and vascularization [29]. Because Hox proteins, TGFβ, HGF, FGF-2 and FGFR1 have important roles in human reproductive development and function, we believe that the evaluation of these growth factors and Hox-positive cells in the human placenta, and correlation with gestational time at delivery and pregnancy outcomes, may provide not only a better understanding of the roles in human development but also may be of clinical importance in reproductive medicine. Thus, the aim of this study was to ascertain the presence of TGFβ, HGF, FGF-2, FGFR1 and HoxB3-positive cells in placentas of different gestational ages, and their association with anthropometric parameters of mothers and newborns.

## 2. Materials and Methods

### 2.1. Study Design and Participants

This was a cross-sectional descriptive study of selected cases. We recruited eligible participants from pregnant women at any gestational age from the 22nd week of pregnancy who were admitted for delivery at the Riga Maternity Hospital. The study was conducted in accordance with the Declaration of Helsinki, and the protocol was approved by the Ethics Committee of the Riga Stradins University (12032009).

The inclusion criteria were human immunodeficiency virus negative, legal age (more than 18 years), no systemic diseases, access to sufficient antenatal care and agreement to participate in the study.

Study participants were divided into two groups: a term study group of 19 cases with term deliveries and a preterm group that consisted of 34 preterm delivery cases from 22 to 36 weeks of pregnancy with various outcomes.

### 2.2. Anthropometric and Clinical Data

Maternal and placental data obtained from medical childbirth records of the Riga Maternity Hospital consisted of maternal age, body weight before pregnancy and height, calculated body mass index (BMI) (BMI = body mass (kg)/height (m^2^)), weight gain during pregnancy, number of previous childbirths and pregnancy terminations, week of gestation at delivery, mode and course of delivery as well as placental weight and outlook. Neonatal data obtained from medical records of neonatal development included gender, birthweight, body length, head and chest circumferences, and calculated ponderal index (PI) (PI = 100 × body mass (g)/height (cm^3^)).

### 2.3. Sample Collection

Immediately after delivery, study materials were taken from three places of the chosen placentas through all the layers of the placental tissues. Two 1 cm × 1 cm samples were in the periphery and one sample was from the central part of the placenta. Samples were then fixed in picric acid-formaldehyde fixation solution [30] and taken to the Institute of Anatomy and Anthropology at Riga Stradins University for processing.

### 2.4. Immunohistochemistry

Tissue immunohistochemical staining for biomarkers identification was done by the biotin-streptavidin method [31], using the following antibodies:

- HoxB3 (rabbit, polyclonal, working dilution 1:100, Santa Cruz Biotechnology, Inc., Dallas, TX, USA),
- TGFβ (mouse, monoclonal, working dilution 1:1000, Abcam, Cambridge, UK),
- HGF (goat, polyclonal, working dilution 1:300, R&D Systems, Wiesbaden, Germany),
- FGF-2 (rabbit, polyclonal, working dilution 1:200, Abcam, Cambridge, UK),
- FGFR1 (rabbit, polyclonal, working dilution 1:100, Abcam, Cambridge, UK).

All antibodies used in research were diluted with Antibody Diluent (code-938B-05, Cell 136 Marque^TM^, Dublin, CA, USA).

Negative and positive controls were provided to avoid background staining and nonspecific binding of secondary antibodies. For negative controls, the primary antibody was omitted during preparations. For positive controls, tissues were used known to contain the molecular factors required either from the manufacturer’s recommendations or from previous research studies in the Institute of Anatomy and Anthropology: brain tissues for TGFβ, six weeks embryo for HGF, fetal liver for FGF-2, umbilical cord for FGFR1 and human Fallopian tube for HoxB3 products.

Immunohistochemistry findings were evaluated semiquantitatively by the extent of staining found in positive cells or extracellular matrix structures in a visual field [32] with the following score criteria: no staining 0, occasional 0/+ (0.5), few + (1), moderate ++ (2), numerous +++ (3), and abundant staining ++++ (4). Evaluations were performed after complete observations of both samples for each placenta.

### 2.5. Statistical Analysis

Descriptive statistics for the entire study group and the selected study groups were performed. For the description of each parameter, mean and standard deviation (SD) were used. For cross-sample mean comparison, inferential statistical Student’s *t*-tests [33] were performed. This method was chosen because of the approximate normality of the data analyzed. To evaluate the cross-compliance of two variables Pearson’s correlation coefficient (r) was calculated [34]. Pearson correlation was chosen due to the normal distribution of the data analyzed. Analyses were performed using Statistical Package for the Social Sciences (SPSS) program version 23.0 (IBM Corporations, Armonk, NY, USA). The reported statistical significance was set at *p* < 0.05, and a statistical significance of *p* < 0.01 was sometimes reported for a more comprehensive evaluation.

## 3. Results

Maternal anthropometric measurements are shown in Table 1. The 53 women in our study who gave birth had a mean age of 29.79 ± 5.6 years (range 18–39 years) as well as different numbers of prior pregnancies and childbirths (Table 1). Gestational age at the time of delivery varied from 22 to 40 weeks. Maternal BMI before pregnancy varied from 17.2 to 36.6 kg/m^2^ with a mean BMI of 23.74 ± 4.72 kg/m^2^.

In the study, maternal anthropometric parameters (weight before pregnancy, body height, BMI before pregnancy, weight gain during pregnancy), evaluated by Pearson two-tail test did not show statistically significant correlations with maternal age, number of pregnancies or deliveries, and the gestational time at delivery. Neonatal birthweight varied from 540 g to 4630 g with a mean weight of 2367.15 ± 1122.59 g. Neonatal birth length varied from 28 cm to 59 cm with a mean length of 45.51 ± 7.433 cm. Mean PI was 2.33 ± 0.31 g/cm^3^ (minimum 1.74; maximum 3.13). Head circumference varied from 22 cm to 39 cm (mean circumference 31.15 ± 4.34 cm) and chest circumference from 20 cm to 37 cm (mean circumference 29.19 ± 4.93 cm). On the whole sample, gestational age was directly proportional to anthropometric parameters: body weight (r = 0.906, *p* < 0.01), body length (r = 0.900, *p* < 0.01), head circumference (r = 0.887, *p* < 0.01) and chest circumference (r = 0.905, *p* < 0.01).

We found no remarkable anomalies in the placentas, which had a mean weight of 448.95 ± 157.503 g ranging from 220 g to 930 g. Using t-tests, we found that term placentas were significantly heavier than pre-term placentas (mean 637.50 ± 91.924 g vs. 401.81 ± 133.54 g respectively, *p* < 0.01).

Next, we evaluated correlations between placental weight and the maternal parameters age, number of prior pregnancies, number of childbirths, maternal weight, maternal length, BMI prior to pregnancy, and weeks of gestation, and found statistically significant positive correlations between placental weight and maternal weight before pregnancy (r = 0.342, *p* = 0.036) and weight gain during pregnancy (r = 0.342, *p* = 0.036). We found a strong correlation between placental weight and week of gestation (r = 0.537, *p* < 0.01).

When comparing the term and preterm placenta groups, we found correlations between placental weight and maternal weight gain during pregnancy in term placentas (r = 0.863, *p* < 0.01) and between placental weight and weeks of gestation in preterm placentas (r = 0.358, *p* = 0.044).

Although we did not find evidence of a correlation in the term placenta group, we found a correlation between placenta weight in the preterm placenta group and several neonatal parameters: body weight (r = 0.606, *p* < 0.01), body length (r = 0.566, *p* < 0.01), head circumference (r = 0.548, *p* = 0.001), chest circumference (r = 0.629, *p* < 0.01).

**Figure 1:**
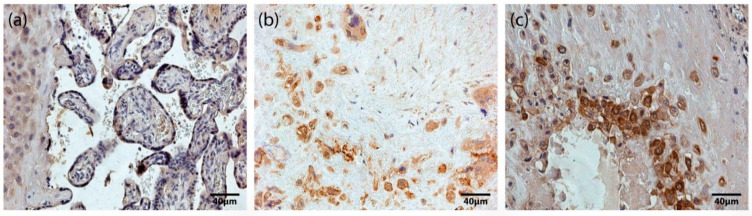
HoxB3-containing structures of the placenta. (**a**) Maternal part and tertiary villi of a 31 gestational week placenta with numerous (+++) HoxB3-containing cells of extravillous trophoblast, cytotrophoblast and syncytiotrophoblast (HoxB3 IMH, X 250). (**b**) Maternal part of a 32 gestational week placenta with numerous (+++) HoxB3-containing Höfbauer cells (HoxB3 IMH, X 250). (**c**) Maternal part of a 40 gestational week placenta with numerous (+++) HoxB3-containing extravillous trophoblast and Höfbauer cells (HoxB3 IMH, X 250).

Based on our immunohistochemistry analysis of all placentas obtained in our study, we found occasional (0/+) to numerous (+++) HoxB3-positive staining in different types of placental cells, specifically cytotrophoblasts, syncytiotrophoblast, extravillous trophoblasts, and Höfbauer cells (Figure 1). In addition, we found no correlation between the number of HoxB3-positive cells and gestational age. Differences between the mean ± SD values of HoxB3-positive cells in the studied fields in term (1.16 ± 0.71) and preterm (1.44 ± 0.72) placentas were not statistically significant.

**Figure 2:**
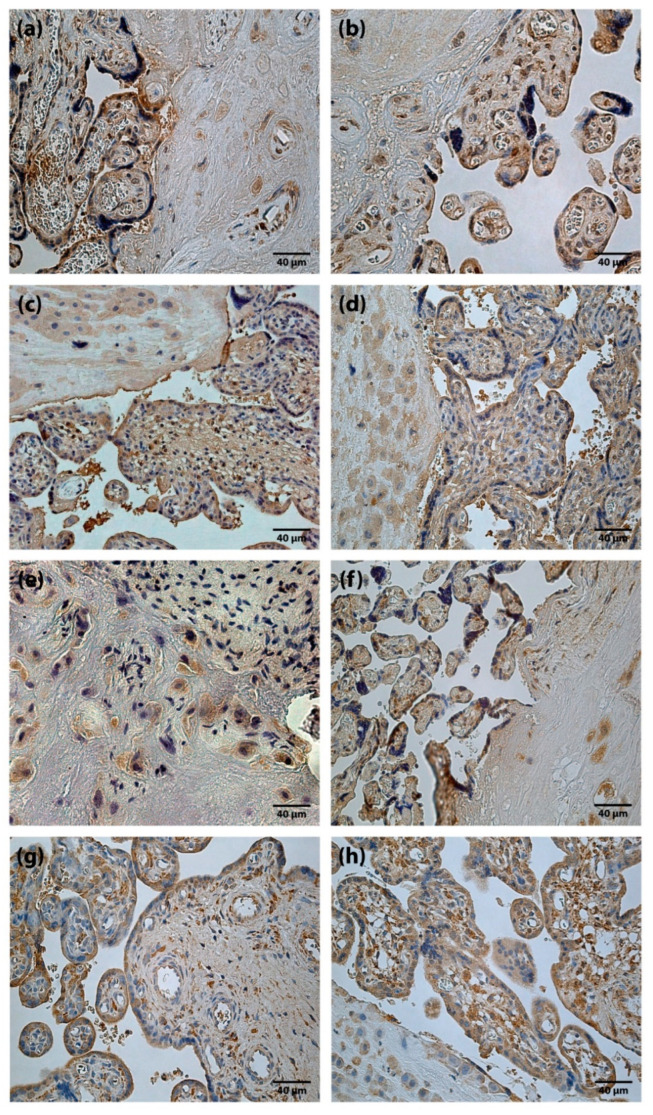
Immunoreactive structures of placenta. (**a**) Moderate amount (++) of TGFβ positive cells in a full-term placenta (TGFβ IMH, X250). (**b**) Moderate amount (++) of TGFβ positive cells in a preterm placenta (TGFβ IMH, X250). (**c**) Abundance (++++) of HGF positive cells in a full-term placenta (HGF IMH, X 250). (**d**) Abundance (++++) of HGF positive cells in a preterm placenta (HGF IMH, X 250). (**e**) Moderate amount (++) of FGF-2 positive cells in a full-term placenta (FGF-2 IMH, X 250). (**f**) Moderate amount (++) of FGF-2 positive cells in a preterm placenta (FGF-2 IMH, X 250). (**g**) Moderate to numerous (++/+++) FGFR1 positive cells in a full-term placenta (FGFR1 IMH, X 250). (**h**) Numerous (+++) FGFR1 positive cells in a preterm placenta (FGFR1 IMH, X 250).

TGFβ findings in placentas were variable, from occasional (0/+) to moderate (++) TGFβ positive structures (Figure 2a,b). Differences between the mean rank values of TGFβ containing cells of the study groups were not statistically significant.

HGF positive cells (cytotrophoblast, syncytiotrophoblast, extravillous trophoblast, Höfbauer cells and cells of extraembryonic mesoderm) appeared from none (0) to abundant (++++) (Figure 2c,d) in the placentas of all gestational ages. Differences between the mean rank values of HGF positive cells of term and preterm placentas were not statistically significant.

FGF-2 positive cells with predominantly weak immunoreactivity were observed. The results of the present study, in some cases, showed moderate (++) number of FGF-2 containing cells (Figure 2e,f). At the same time, FGFR1 containing cells were detected from occasional (0/+) to abundant (++++) (Figure 2g,h) in a visual field. Differences between the mean rank values of FGF-2 and FGFR1 positive cells in the placentas of the study groups were not statistically significant.

Correlating the rank values of the growth factor and receptor-positive cells in placentas among themselves, with gestational age of placenta and with maternal parameters in the whole study, we did not find statistically significant correlations. Evaluating correlations of the rank values of the growth factor and receptor positive cells with neonatal parameters in the whole study the rank value of HGF showed a negative correlation with neonatal head circumference.

When comparing the appearance of HoxB3 staining with growth factors and FGFR1-positive cells, gestational age and maternal parameters in placentas of the whole study, as well as in term and preterm placentas, we found no significant correlation. However, correlating the appearance of HoxB3 staining in placentas of the whole study group with neonatal parameters, we found a statistically a significant negative correlation with PI (r = −0.323, *p* = 0.018). In addition, we found a positive correlation with the appearance of HoxB3 staining and neonatal body length (r = 0.541, *p* = 0.046) in the term placenta group, and a negative correlation PI (r = −0.461, *p* = 0.008) in the preterm placenta group.

## 4. Discussion

The placenta, a temporary organ responsible for transport functions between maternal and fetal circulation during intrauterine development, has been shown to influence birthweight [35]. In the current study, we detected positive correlations between placental weight and maternal weight before pregnancy and weight gain during pregnancy. We also found a strong correlation between placental weight and week of gestation, which was directly proportional to fetal anthropometric parameters (body weight, body length, head circumference, chest circumference). Moreover, a high placental weight to fetal body weight ratio is correlated with short-term adverse perinatal outcomes [35]. Placental weight is an important determinant of both birthweight and fetal growth [36].

Formation of the interface between maternal and fetal circulations is controlled by several growth factors, cytokines and transcription factors including homeobox genes [5]. Hox proteins have been associated with different fields of human medicine. The HoxB3 was identified as a potential biomarker for osteoarthritis [37] and might be used for the prediction of clinically isolated syndrome progress to relapsing-remitting multiple sclerosis [38,39]. The aberrant expression of HoxB3 might promote Parkinson’s disease via dysregulating sphingolipid and glutathione metabolism [40].

Evaluating the appearance of HoxB3-positive cells in placentas from preterm and term groups, we found slightly more HoxB3-positive cells in the preterm placentas, although the difference was not statistically significant. Chen et al. reported that overexpression of HoxB3 promoted cell apoptosis but inhibited cell proliferation, migration, and invasion [7]. We found HoxB3 staining in various amounts in all placentas from this study, indicating it also has a certain role in the development of the maternal-fetal unit.

The TGFβ family establishes the fetal-maternal interface and is a vital regulator of placental development [41] and fetoplacental angiogenesis [42]. TGFβ exerts several modulatory effects on trophoblast cells, such as inhibition of proliferation and invasiveness, and stimulation of differentiation by inducing multinucleated cell formation [21]. Interestingly, in our analysed specimens, there were very few or occasional TGFβ-positive structures detected. It is reported that the TGFβ levels drop from second to third trimester in pregnancies, where the fetus size is appropriate-for-gestational age [43]. This finding might be of importance pointing out TGFβ role also in the modulation of fetal growth.

There seemed to be less HGF positive structures in placentas of more advanced gestational age; however, no statistically significant correlation was found. The tendency of a decreasing number of HGF-positive cells seems to depend on the adaptation/ageing of placenta. In the literature, there are studies suggesting that HGF promotes regeneration of tissues [44,45]. HGF inhibits apoptosis in the placentas of pathological pregnancies, e.g., in case of pre-eclampsia [46,47].

FGF-2 is expressed by the trophoblast and endothelial cells in placentas of humans [48]. FGF-2 takes part in placental angiogenesis [49] and influences trophoblast cell behavior [50]. Expression of FGF2 increases exponentially in fetoplacental tissues in late ovine gestation [48]; however, in the current study, weak FGF-2 immunoreactivity in the human placenta was observed. One study described that FGF-2 is highly expressed in the first trimester; the increased expression of FGF-2 may promote pathological angiogenesis [49]. Several studies have associated increased expression of FGF and FGFR with pathologies in pregnancies such as preeclampsia [51] or IUGR [49]. Additionally, it could be assumed that the less detected FGF-2 and more prominent FGFR1 finding points out a compensatory receptor stimulation.

In our study, we found evidence of correlations between the appearance of HoxB3-positive cells in placentas and maternal and neonatal parameters. In the term birth cases, we found a significant positive correlation between the appearance of HoxB3 staining in the placenta and neonatal body length. It is depicted in the literature that the Hox genes are an evolutionary conserved family of genes that control the anterior-posterior axis and dorsal-ventral anatomic development during embryogenesis [3]. Posterior Hox genes may control axial elongation rate by progressively down-regulating the Wnt/βcatenin pathway [52]. The development and growth of the fetus are determined by the exchange of nutrients through the placenta during pregnancy. HoxB3 is a major regulator of angiogenesis [19], which makes us speculate that HoxB3 may impact placental status and, therefore, fetal growth, too.

The limitations of our study are associated with a relatively isolated gene analysis. Possibly, an impact of other gene proteins/ transcription factors on placental status could be observed, which we plan to investigate in the future. This time, HoxB3 was studied in context with the expression of TGFβ, FGF-2, FGFR1 and HGF to find out their potential role in placental status and fetal growth. It has to be taken into account that this was a hospital-based study, so its results may not be applicable on the whole population of Latvian pregnant women. In this study, only the immunohistochemistry method was used, however, but it could be valuable to compare our results applying other methods such as Western Blot, PCR and ELISA in the future.

## 5. Conclusions

The number of HoxB3-positive cells persisting in all post-delivery placentas from 22 weeks of gestation did not correlate with gestational age but with neonatal anthropometrical indices, indicating the role of HoxB3 not only in placental development, but also in the longitudinal growth of the fetus.

HGF and FGFR1 positive structures were seen in all the placental samples, suggesting a permanent growth ability of the organ. TGFβ and FGF-2 did not play a significant role in the development of the placenta beyond the 22nd week of pregnancy while FGFR1 immunoreactive cells increase with advancing gestation, indicating increasingly evolving maturation (growth, proliferation) of the placenta, especially in the third trimester.

## Figures and Tables

**Table 1:** Maternal data.

Parameter	Minimum	Maximum	Mean ± Standard Deviation
Weight before pregnancy (kg)	46	109	65.82 ± 12.75
Body height (cm)	150	182	166.33 ± 7.025
BMI before pregnancy (kg/m^2^)	17.20	42.60	23.74 ± 4.74
Weight gain during pregnancy (kg)	0	26	10.90 ± 5.81
Pregnancy	1	7	2.59 ± 1.64
Delivery	1	6	1.75 ± 0.98

## Data Availability

The data used in this study are available from the corresponding author upon request.
